# Quality improvement as a framework for behavior change interventions in HIV-predisposed communities: a case of adolescent girls and young women in northern Uganda

**DOI:** 10.1186/s12981-018-0190-2

**Published:** 2018-01-25

**Authors:** Esther Karamagi, Simon Sensalire, Juliana Nabwire, John Byabagambi, Alfred O. Awio, George Aluma, Mirwais Rahimzai, Jacqueline Calnan, Sheila Kyobutungi

**Affiliations:** 1 University Research Co., LLC, USAID Applying Science to Strengthen and Improve Systems (ASSIST) Project, USAID, Kampala, Uganda; 2 United States Agency for International Development (USAID), Kampala, Uganda

**Keywords:** HIV risk, Quality improvement, Model, Behavioral change

## Abstract

**Background:**

Despite the conventional approaches to HIV prevention being the bedrock for early reductions in HIV infections in Uganda, innovations that demonstrate reduction in risk to infection in vulnerable populations need to be embraced urgently. In the past 2 years, a USAID-funded project tested a quality improvement for behavior change model (QBC) to address barriers to behavioral change among adolescent girls and young women (AGYW) at high risk of HIV infection. The model comprised skills building to improve ability of AGYW to stop risky behavior; setting up and empowering community quality improvement (QI) teams to mobilize community resources to support AGYW to stop risky behavior; and service delivery camps to provide HIV prevention services and commodities to AGYW and other community members.

**Methods:**

We recruited and followed a cohort of 409 AGYW at high risk of HIV infection over a 2-year period to examine the effect of the QBC model on risky behaviors. High-risk behavior was defined to include transactional sex, having multiple sexual partners, and non-use of condoms in high-risk sex. We documented unique experiences over the period to assess the effect of QBC model in reducing risky behavior. We analyzed for variances in risk factors over time using repeated measures ANOVA.

**Results:**

There were statistically significant declines in high-risk behavior among AGYW over the QBC roll-out period (p < 0.05). Univariate analysis indicated reduction in AGYW reporting multiple sexual partners from 16.6% at baseline to 3.2% at follow up and transactional sex from 13.2 to 3.6%. The proportion of AGYW experiencing sexual and other forms of gender based violence reduced from 49% a baseline to 19.5% at follow up due to the complementary targeting of parents and partners by QI teams.

**Conclusion:**

The QBC model is appropriate for the context of northern Uganda because it provides a framework for the community to successfully drive HIV prevention efforts and therefore is recommended as a model for HIV prevention in high-risk groups.

## Background

This paper highlights the problem of risky sexual behavior that present a greater risk to HIV infection and transmission of HIV among adolescent girls and young women (AGYW). We use the terms ‘adolescent girls and young women’ to refer to ages 10–24 being the target group of an evidence based behavioral intervention. The effect of a quality improvement for behavior change (QBC) model on reducing risky sexual behavior among a high risk population of AGYW in northern Uganda is explained.

AGYW in the post conflict northern Uganda experience at least three or more HIV risk factors every day. They are victims of gender-based violence including rape, experience cross generational sex, engage in transactional sex, have multiple sexual partnerships, live in absolute poverty-stricken households, are grossly illiterate, and abuse drugs. The risk is even higher with changing individual-level behaviors that characterize AGYW as they transition from childhood to adulthood. The risk of exposure to various risky behaviors even increases further in societies with diminished stability [1].

According to the AIDS indicator survey [2], northern Uganda has HIV prevalence of 10.1% among women aged 15–49 and HIV prevalence among young people is markedly higher among young women than among young men and women become infected at younger ages than men [2]. Sexual transmission accounts for 76% of new HIV infections and is partly attributed to inconsistent and incorrect use of condoms, casual sex, multiple sex partners and extramarital sex [2]. The risk of acquiring HIV applies to AGYW with multiple partnerships, as well as to those with male partners who have (or have had) multiple partners [2]. Vulnerability to HIV infection is especially higher during forced or coercive sex due to the increased chance of vaginal tearing, especially in younger women whose vaginas are not mature. In the extreme circumstances, family members and partners are the perpetuators of violence on the victims including rape [3]. The World Health Organization (WHO) reports that globally, over one-third of women have experienced some form of physical or sexual violence [3].

A critical constraint experienced by young people wishing to practice safer heterosexual intercourse consists of the degree of control they have within sexual encounters. In general, young women have far less control over their sexual encounters than do young men. One obvious way power is manifest in heterosexual relationships is through the presence or threat of violence against young women by young men. Over the past decade strong evidence has emerged on the relationship between partner violence and HIV. There is equally strong evidence for and recognition of successful community strategies to prevent intimate partner violence and vulnerability to HIV [4]. In northern Uganda, for example, the legacy of violence due to political conflict embedded violence as a normal part of gendered relation including rape.

The exploration and formation of identity is considered by many to be the primary developmental goal of adolescence. Socially, adolescents are searching for a sense of belonging from peers, who influence behavior. These milestones occur during a period of decreased adult supervision when young people still have limited knowledge, self-confidence and life skills, which can lead to engagement in behaviors that heighten HIV risk [5]. In the context of HIV prevention, it is essential for parents to be aware of the unique biological, social and emotional challenges of adolescence in order to skillfully help their child navigate their way through adolescence to adulthood, in addition to meeting their financial needs. Optimal parenting requires flexibility and adaptability to meet the particular needs of an individual child or adolescent in a developmentally-appropriate manner. Several studies have demonstrated a link between young people’s sexual behavior and levels of parental monitoring and parent–child communication in Western countries. However, little is known about this association in African settings, especially among young people living in high poverty settings [6].

Adolescent behavior is influenced by family, peers, school, neighborhood, and the broader socio-cultural context [7]. Parents are positioned to assume a central role in protecting against adolescent involvement in risk behaviors [8]. Parents may exert their influence on adolescent risk behaviors through different pathways including parent-adolescent communication and parental monitoring [9]. Studies have shown that parent-adolescent communication about sexual behavior especially communication before the onset of youth sexual activity promotes healthy sexual decision-making and decreases adolescent involvement in sexual risk behaviors [10].

## The intervention

Many interventions focus on changing individual-level behaviors rather than addressing the larger contextual and structural landscape within which young people live. In response to this concern, USAID ASSIST Project rolled out the QBC model using the community-based quality improvement (QI) teams under the “Determined Resilient Empowered AIDS-free Mentored and Safe” (DREAMS) initiative in the post-conflict northern Uganda districts of Gulu and Omoro (Acholi sub region) and Oyam and Lira (Lango sub region).

The regions was a battleground for the Lord’s Resistance Army (LRA), also known as the Lord’s Resistance Movement, a rebel group which operated in northern Uganda for more than 2 decades and later in South Sudan, the Central African Republic, and the Democratic Republic of the Congo. It was originally known as the United Holy Salvation Army and largely functioned as a personality cult of its leader Joseph Kony a self-declared prophet. The rebel activities were characterized by human rights violations, including murder, abduction, mutilation, child-sex slavery, and forcing children to participate in hostilities [11]. As a result, the population in the region lived in concentrated camps and inhumane conditions that facilitated HIV infection and transmission.

DREAMS is a multi-country initiative supported by the United States President’s Emergency Plan For AIDS Relief (PEPFAR) that brings together a combination of interventions already known to prevent HIV among AGYW [12]. DREAMS targets AGYW in ten Sub-Saharan African countries where nearly half of all new HIV infections occurred among AGYW globally in 2014 [12]. However, the success of known interventions is affected by the model of delivery used to guarantee the right target group receives all components of the intervention [12].

The United States Agency for International Development (USAID) Applying Science to Strengthen and Improve Systems (ASSIST) Project tested the QBC model over a 2-year period among a high-risk population of AGYW that experience at least three or more HIV risk factors in life such as gender-based violence (GBV) including rape, cross-generational sex and early marriage, transactional sex, multiple sexual partnerships and drug abuse [13]. The model comprises three core interventions: (1) skills building and peer to peer support in topics such as saying no to sex and negotiating condom use to improve the ability of AGYW to stop risky behavior and to influence safer sex practices with their partners; (2) setting up and making functional community QI teams to mobilize community resources to support the young women and their partners to stop risky behavior; and (3) holding service delivery camps to provide HIV prevention services and commodities to AGYW, their partners and other community members mobilized by the community QI teams.

The QI team members were local community members from the same cultural and social context as the AGYW target population, including community elders, religious leaders, peer facilitators, local council members, Village Health Teams (VHTs), health workers, and teachers. Peer facilitators were AGYW from different communities that facilitated skills building and peer to peer support sessions and used their life experiences to relate to AGYW in similar circumstances. Most at risk AGYW were identified using ‘The Girls Roster toolkit.’ The toolkit is a practical tool to help increase girls especially poor adolescent girls’ access to vital resources, facilities, and services. It helps in understanding the full universe of girls in a program catchment area; breaking them into meaningful segments by age, by schooling, and by marital, childbearing, and living-arrangement status; and, through appropriate community engagement, increasing girls’ access to a fair share of community resources.

The USAID Strengthening Decentralization for Sustainability (SDS) project supported the processes of training community resource persons to use the tool and to map priority communities. The ASSIST project randomly selected 12 of the priority communities to demonstrate implementation of QI for HIV prevention using the QBC model. Within the 12 communities all the AGYW mapped and registered using the Girls Roster tool were engaged in the ASSIST supported activities. QI teams identified AGYW in their communities by their various risks such as gender-based violence (GBV) including known rape victims, cross-generational sex and early marriage, transactional sex, multiple sexual partnerships and drug abuse. Teams worked with AGYW to develop risk reduction plans consonant with the norms, culture, and aspirations of the target population and followed up with the AGYW to support behavioral change using a set of messages that were tailored to the specific needs of the AGYW. Participatory approaches to behavioral change communication were used, such as sharing of experiences, problem identification, and personalized risk reduction plans.

QI teams worked with facility teams to create demand for HIV prevention services through quarterly integrated health service camps for AGYW and partners of young women to improve access to a standard care package for HIV prevention including HIV counseling and testing (HCT), screening and treatment of sexually transmitted infections (STIs), family planning services of their choice, condom awareness and distribution, risk reduction counselling, and provision of tetanus toxoid to partners in preparation for voluntary medical male circumcision (VMMC). According to Ministry of Health [2], HCT is the entry point to HIV prevention. There is also an epidemiological relationship between STIs and HIV infection. A study conducted by Sensalire et al. [14] showed that awareness of HIV prevention through the use of condoms increased the likelihood of use by four times among adolescents. According to the World Health Organization [15], medical male circumcision reduces the risk of female-to-male sexual transmission of HIV by approximately 60%.

Quality improvement teams also targeted parents of all AGYW irrespective of whether they were minors or not. Several studies have demonstrated a link between young people’s sexual behavior and exposure to information and levels of parental monitoring and parent–child communication in Western countries [6]. In the context of a behavior change intervention, it was essential for parents to be aware of the unique biological, social, and emotional challenges of adolescence in order to skillfully help their child navigate their way through adolescence to adulthood, in addition to meeting their financial needs. The involvement of parents under the intervention was based on the hypothesis that good parenting reduces sexual risk behavior among adolescents. QI teams mobilized parents and caregivers of AGYW for parenting training sessions with the aim of empowering them with knowledge and skills to better engage and communicate with their children and support them reduce their risk of HIV sustainably.

In addition to AGYW and their parents, the QI teams also involved partners of young women. They were mobilized for partners’ meetings by the young women themselves and partly by the QI teams in instances where the partner was known to them by virtue of being community members in their close proximity. In some instances, partners are the perpetuators of violence on the victims, including rape and at baseline 10% of the AGYW had experienced sexual violence from partners. QI teams held meetings with partners to identify risk factors and mechanisms to address them. The partner meetings addressed the risk of multiple sexual partners, unprotected sex, domestic violence of all forms, unwanted pregnancies, social and financial empowerment of partners and repugnant norms that risk to HIV infection. Partners therefore, became change agents in the communities.

A critical constraint experienced by young people wishing to practice safer heterosexual intercourse consists of the degree of control they have within sexual encounters. In general, young women have far less control over their sexual encounters than do young men. One obvious way power is manifested in heterosexual relationships is through the presence or threat of violence against young women by male partners [16]. Partner violence increases the risk of HIV, and there is strong evidence for and recognition of successful community strategies to prevent intimate partner violence and vulnerability to HIV [16]. In northern Uganda, due to the prolonged political conflict, GBV, including rape, is common. This intervention recognized the connection between HIV infection and gender violence and therefore, involved partners and parents.

## Methods

### Study subjects

The study cohort comprised of transactional sex workers, AGYW with multiple sexual partners and cross-generational sex, drug users, and victims of gender-based sexual violence and had reported to have dropped out of school.

### Study design

We utilized a longitudinal cohort evaluation design among a cohort of 409 AGYW. The same cohort was then followed after the roll-out of QBC interventional model by QI teams over three time periods (follow-up assessments) to assess changes in risky behaviors. The assessments largely determined the effect of the model on risk reduction among AGYW known to be at risk. We documented QI activities alongside evaluations of AGYW behavior and status to validate findings about what worked in achieving reduction in risk behavior. All 409 AGYW had not been exposed to any intervention at the time of the baseline.

### Data collection

Baseline and three follow-up assessments collected key information on risky sexual behavior such as multiple sexual partnerships, transactional sex, non-use of condoms in risky sex, gender-based sexual violence, unwanted pregnancy, and self-efficacy to negotiate for safer sex aimed at assessing the effect of skills building on behavioral change. The studies were spaced by 3 months after the baseline because of the peer and inter personal approach to behavior change being likely to influence the nature of risk behavior being addressed. A standard questionnaire was administered to the cohort at each study period so as to maintain consistence in the behaviors being tracked. The sample size distribution for the longitudinal studies is shown in Table 1.

**Table 1:** Demographic, socio-economic characteristics and sample distribution of AGYW over the study periods

Sample characteristic	Baseline	Follow up 1	Follow up 2	Follow up 3	Sig
Study sample	409	409	408	394	na
Age categorization					
10–14	9 (37)	9 (37)	6.4 (26)	1.3 (5)	0.000
15–19	42.8 (175)	42.8 (175)	38.2 (156)	43.7 (172)	
20–24	48.2 (197)	48.2 (197)	55.4 (226)	51.0 (201)	
25 +	0	0	0	4.1 (16)	
Marital status					
Single with no partner	28.1 (115)	28.1 (115)	22.8 (93)	12.7 (49)	0.000
Single with a partner	41.8 (171)	41.8 (171)	40 (168)	42.6 (168)	
Married	22 (90)	22 (90)	30.6 (125)	36.3 (143)	
Divorced/separated	7.8 (32)	7.8 (32)	6.4 (26)	8.6 (34)	
Widowed	0.2 (1)	0.2 (1)	0.2 (1)	0	
Education					
None	1.7 (7)	7 (1.7)	0	0.8 (3)	0.211
Primary	62.6 (256)	62.6 (256)	65 (256)	67.5 (266)	
O level	30.6 (125)	30.6 (125)	27.9 (114)	27.4 (108)	
A level	2.2 (9)	2.2 (9)	4.4 (18)	2.8 (11)	
Occupation					
Student	9.5 (39)	9.5 (39)	10.3 (42)	4.8 (19)	0.000
Unemployed/looking for work	22 (90)	22 (90)	22.3 (91)	32.7 (129)	
Doing personal business	26.4 (108)	26.4 (108)	28.2 (115)	30.5 (120)	
Agriculture	26.9 (110)	26.9 (110)	27.2 (111)	23.6 (93)	
Employed	1.5 (6)	1.5 (6)	2.9 (12)	3.8 (15)	
Other	29.9 (54)	29.9 (54)	17 (37)	8.7 (31)	

*na* not applicable

There was one participant lost to follow-up at period 2 and 15 lost to follow-up at period 3. All 16 were lost because they moved out of the target communities, to new places of residence, and could not be traced. We collected data during follow-up on the update of HIV risk reduction services offered during camps, including HCT, STI screening and treatment, family planning services of one’s choice, condom awareness and distribution, risk reduction counseling, and provision of tetanus toxoid to partners in preparation for VMMC.

The purpose of the study was explained first to the parents. Parental consent was obtained for minors. Individual assent and consent was then obtained from each study subject. In both cases, the consent process involved highlighting the study objective, methods, risks and benefits of participation, the confidentiality of data, and voluntariness of participation to the study. Three digit codes were used as unique identifiers other than names to protect the identity of participants. There were no refusals to participate in the studies from both parents and AGYW to the extent that even the newly recruited AGYW wished to be enrolled in the studies.

The questionnaire had different domains covering risky sexual behavior, self-efficacy, exposure to HIV information, gender-based violence, uptake of services, parenting, and partner support. All aspects of the assessment were pretested in the non-intervention communities by the investigating team prior to the baseline study. The pretest involved interviews with AGYW to assess clarity, wording, understandability of questions, duration of interview and appropriateness of field procedures and logistics. The lessons learned from the pretest were used to finalize the survey instruments and logistical arrangements for the baseline study.

### Ethical considerations during data collection

Ethics approval was obtained from a locally approved Institutional Review Board (IRB) and the Uganda National Council of Science and Technology (UNCST) under Reference number REC REF 0201-2017. All consent forms had contacts of the study team and of the chairperson of the IRB in case participants had concerns about the study or in case they needed to know their rights, respectively. Administrative clearances were obtained in the districts and from the community leaders within the intervention areas. Participation was entirely voluntary, and consent was based on full information about the study and intervention.

### Data analysis

Each set of variables was analyzed and presented for both baseline and follow-up periods except in as far as they apply to baseline or follow-up only. Univariate analysis explored each of the variables of interest separately so as to obtain comparative statistics. Analysis of Variance (ANOVA) was used to determine any difference over time that would produce an overall level of confidence that is very close to the level desired (95%). To determine whether any of the differences between the means were statistically significant, we compared the *p* value to the significance level to assess the null hypothesis at an alpha 0.05.

## Results

### Description of the cohort over the study period

There were shifts in participants’ ages over the study period because of the continuous nature of the variable. The change was statistically significant at p = 0.000. There were changes in marital status over the period as AGYW in the age group 20–24 got married. Education levels did not change over the period because study cohort comprised of predominantly school drop outs. Statistical differences were noted in the occupation of the AGYW, with a positive change being noticed in the proportion of AGYW engaged in self-employment. The various proportions of the characteristics of AGYW and their corresponding numbers (n) are shown in Table 1.

### Changes in risky sexual behavior

There was a reduction in risky sexual behavior among the AGYW exposed to the QI for behavior change model (Table 2). High-risk sex was operationally defined to include: multiple sexual partners, transactional sex, and unprotected sex with a non-regular partner. Prior to the formation and activation of QI teams, 16.6% (n = 66) of AGYW out of the 409 had multiple sexual partners. Only 23.5% (n = 15) of AGYW with multiple sexual partners at baseline reported consistent use of condoms during this high risk sex. With the formation and activation of QI teams, the proportion of AGYW reporting multiple sexual partnership declined to 3.2% (n = 12) out of the 394 AGYW. The statistically significant decline in the proportion of AGYW reporting multiple sexual partners (p = 0.000) was attributed to the behavioral change activities of the QI teams in the DREAMS communities. These results are shown in Table 2.

**Table 2:** Variations in sexual behavior practices over the study period

High risky sex among AGYW In the past 3 months	Baseline % (n)	Follow up 1 % (n)	Follow up 2 % (n)	Follow up 3 % (n)	Sig
Had multiple sexual partners	16.6 (66)	11.4 (33)	5.2 (17)	3.2 (12)	0.000
In transactional sex	13.2 (54)	7.1 (15)	3.1 (10)	3.6 (14)	0.051
Had protected sex with multiple sexual partners	23.5 (16)	60.6 (20)	69.1 (9)	75 (9)	0.042
Had protected sex in transactional sex	29.6 (16)	71.4 (20)	70 (7)	57.1 (8)	0.573

We further determined engagement in transactional sex before the formation and activation of QI teams. In Table 2, we found that 13.2% (n = 54) out of 409 AGYW at baseline were engaged in transactional sex and yet only 29.6% (n = 15) out of the 54 AGYW in transactional sex at baseline used condoms at every sexual encounter during transactional sex. With the activation of QI teams, there was a significant decline (p = 0.003) in the proportion of AGYW in transactional sex to 3.6% (n = 14) out of 394 AGYW at follow-up 3 and an increase in consistent use of condoms to 57.1% among AGYW in transactional sex following the intervention.

### Parents and partner interventions

As positive behavior change became apparent, three new interventions were introduced and rolled out by QI teams in the course of the intervention, namely: partner support, parent support, and addressing gender based violence (GBV). QI teams organized meetings and educated sexual partners of the young women on HIV risky behaviors and prevention within the DREAMS communities. The meetings addressed issues affecting male sexual partners support to AGYW HCT, dangers of multiple sexual partners and inconsistent condom use. Parenting sessions within communities by QI teams aimed at supporting parents/guardians to improve parenting skills and support their children to reduce risk of HIV infection.

### Parental and partner support

We explored any material, financial, health or psychosocial support given to the AGYW by their parents and partners of young women at follow-up 3 following the expansion of the intervention beyond the AGYW. As a midterm intervention, we documented parental and partner support 3 months into the intervention to determine whether or not this QI strategy of the QI teams had yielded results in a short run.

Following the intervention involving parents and partners of AGYW between follow up 2 and 3 (3 months) by QI teams through parents and partner meetings to address HIV risk factors, we found that the proportion of AGYW receiving parental support increased from 68.1% (n = 278) out of 408 AGYW to 72.6% (n = 286) out of the 394 AGYW, and partner support from 56.1% (n = 229) out of 408 AGYW to 82.5% (n = 325) out of the 394 AGYW. The results are shown in Table 3.

**Table 3:** Parenting and partner support for the AGYW

Intervention	Follow up 2		Follow up 3	
	Percent	Number	Percent	Number
Received any form of support from parent				
Yes	68.1	(278)	72.6	(286)
No	31.9	(130)	27.4	(108)
Received any form of support from partner				
Yes	56.1	(229)	82.5	(325)
No	27.2	(180)	17.5	(69)

### Effect of parental support on sexual behavior

A further analysis was done at follow-up 3 following the involvement of parents and partners to determine the effect of this intervention on sexual behavior of AGYW, with the hypothesis that some parents/guardians and partners are the perpetrators of risky sexual behavioral practices that expose AGYW to the risk of HIV. We found that AGYW who had received parental support of any form (material, financial, health or psychosocial support) engaged in less transactional sex following the intervention (57.1%) compared to those who received no support from the parents. Accordingly, there was a statistical association between the support of parents and transactional sex activity (p = 0.023). The results are illustrated in Table 4.

**Table 4:** Effect of parenting on sexual behavior practices of AGYW by follow up 3

Sexual behavior	Percentage		Sig
	Before parenting intervention	After parenting intervention	
Had multiple sexual partners			
Yes	61.2	58.3	0.292
No	38.8	41.7	
Had transactional sex			
Yes	67.7	42.9	0.023
No	32.3	57.1	
Used condoms consistently with multiple sexual partners			
Yes	54.3	55.6	0.735
No	45.7	44.4	
Used condoms consistently during transactional sex			
Yes	36.8	37.5	0.896
No	63.2	62.5	

### Influence of partner support on sexual behavior

QI teams targeted partners of young women with a behavioral change package addressing GBV, respect and dignity, and HIV risk factors, all aimed at reducing multiple sexual partners, transactional sex and inconsistence in condom use and gender-based violence. The analysis of the influence of partner support on sexual behavior practices of AGYW, shown in Table 5, indicated that within a space of three (3) months, AGYW who received support of their partners engaged less in transactional sex (p = 0.002). This was attributed to the interventions on partners by QI teams.

**Table 5:** Influence of partner support on sexual behavior practices of AGYW by follow up 3

	Percentage		Sig
Sexual behavior	Before parenting intervention	After parenting intervention	
Had multiple sexual partners			
Yes	51.2	52.9	0.461
No	48.8	47.1	
Had transactional sex			
Yes	53.7	20.0	0.002
No	46.3	80.0	
Used condoms consistently with multiple sexual partners			
Yes	16.1	22.2	0.071
No	83.9	77.8	
Used condoms consistently during transactional sex			
Yes	25.6	28.6	0.880
No	64.4	71.4	

### Gender based violence and actions taken by victims

**Fig. 1:**
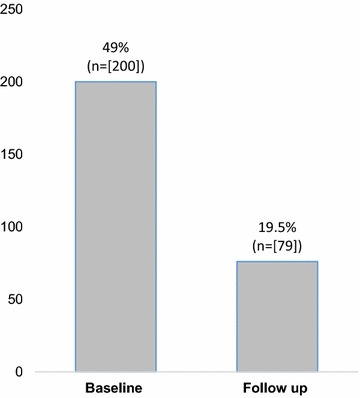
Number of AGYW exposed to domestic violence

QI teams targeted gender-based violence by promoting, respect and dignity, and highlighting HIV risk factors associated with GBV. The proportion of AGYW experiencing violence prior to the intervention out of the 409 AGYW studied at baseline, was as high as 49.1%, but reduced to as low as 19.5% at 3rd follow-up within the same cohort. The findings are illustrated in Fig. 1.

### Actions taken by the victims of violence

**Fig. 2:**
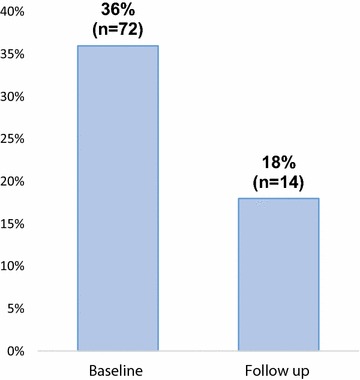
Proportion of AGYW that took action against perpetrators of violence

We investigated the actions taken by the victims of violence on their perpetrators. In Fig. 2, out of the 49.1% (n = 200) of AGYW who were exposed to violence at baseline, 36% kept silent compared to 18% at follow-up 3. The program promoted integrated GBV at follow up to address HIV risk behavior associated with violence. AGYW were encouraged to report to report to local council, parents/guardians, police, or seek medical care when faced with violence.

### Other findings

**Fig. 3:**
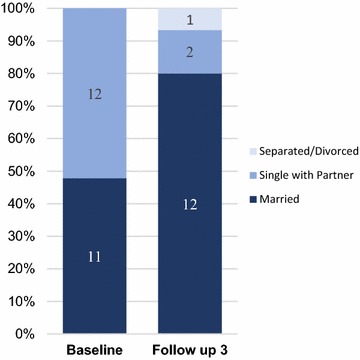
Marital status of the pregnant AGYW

Alongside the need to reduce risky sexual behavior was the reduction of unwanted pregnancy, another outcome of risky sex among the target group. Prior to the intervention, we found that 23 out of the 409 AGYW were pregnant. They were mostly single with a partner and predominantly teenagers (Fig. 3). At follow-up 3, the number of pregnant AGYW declined to 15, and they were mostly married young women aged 20 and above. The QI teams promoted use of condoms and contraceptives during the intervention period.

**Fig. 4:**
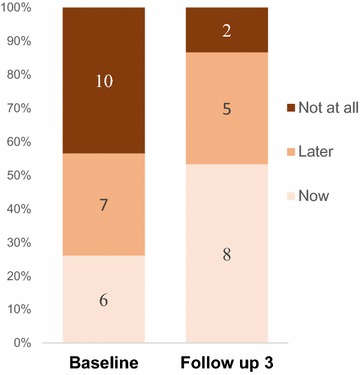
Desired timing of pregnancy of AGYW

We further explored the intentions of pregnant AGYW at the time of conception and found out that 10 out of the 23 pregnancies at baseline were undesired. At follow up, the number of pregnancies declined to 15 and were mostly desired (n = 8) and mostly occurring among married young women aged 20 years and above. Results are illustrated in Fig. 4.

## Discussion

The aggregate effect of radical and sustained behavioral changes in a sufficient number of individuals potentially at risk is needed for successful reductions in HIV transmission. Second, the effect of behavioral strategies could be increased by aiming for many goals (e.g., delay in onset of first intercourse, reduction in number of sexual partners, increases in condom use, etc.) that are achieved by use of multilevel approaches (e.g., couples, families, social and sexual networks). Third, reductions in HIV transmission need a mix of communication channels to disseminate messages to motivate people to engage in a range of options to reduce risk [17].

The QBC intervention addressed multiple sexual partners, inconsistent use of condoms in high risk sex and transactional sex among AGYW through skills building and peer to peer support to abstinence, negotiating condom use; setting up QI teams and holding service delivery camps to provide HIV prevention services and commodities to AGYW, their partners and community mobilization. These multilevel approaches following the intervention in target population, resulted into a significant decline in risky sexual behavior, which suggests the appropriateness of the QBC model in addressing HIV risk in similar settings.

Having multiple sexual partners and transactional sex is one of the important behavioral HIV risk factors that were addressed using the QBC model intervention. Studies have shown that frequent change of sexual partners is associated with higher HIV risk and HIV incidence among young sex workers and sexually exploited adolescent girls remains high in many settings in Sub-Saharan Africa [18]. The study found that transactional sex and multiple sexual partnerships was at 23 and 17% at baseline respectively. In order to lower the risk of HIV by reducing the number of sexual partners, a better understanding of the factors which affect young women’s risk behaviors is needed [19]. Thus peer to peer approach was used to determine the driving factors for multiple sexual partnerships and transactional sex so as to deploy socially and contextually appropriate behavioral change interventions. This resulted into a reduction in the number of AGYW engaging in risky sexual behaviors as compared to the baseline, thus suggesting a need for mix of individual and contextual approaches to effectively tackle the risk of HIV among high risk AGYW.

We investigated exposure to violence by AGYW and found that 10% had been exposed to sexual violence by their partners. In northern Uganda, the legacy of violence due to political conflict embedded violence as a normal part of gendered relations, including rape. Studies have shown that a critical constraint experienced by young people wishing to practice safer heterosexual intercourse consists of the degree of control they have within sexual encounters. Young women have far less control over their sexual encounters than do young men and experience sexual violence [4]. The intervention therefore, targeted partners of the AGYW midway the intervention. The decline in sexual violence to 2.3% and other forms of violence following the intervention among male partners suggest, that male partners can be agents of behavioral change themselves. It also suggests that targeting AGYW alone without their partners may not eliminate partner-driven risk behavior. This suggests complementary interventions that focus both young girls and their partners are more effective than interventions that target only AGYW.

Parents have the potential to protect against adolescent sexual risk, including early sexual behavior, inconsistent condom use, and outcomes such as pregnancy and STIs. Identification of the specific parenting dimensions associated with sexual risk in adolescence and young adulthood is necessary to inform and focus prevention efforts [20]. We found that engaging parents/guardians of the AGYW resulted in positive behavioral changes, notably a reduction in transactional sex. There was a statistical association between parental support and the reduction in the proportion of AGYW in transactional sex. Parental support was mostly in the form of financial support to the AGYW. This underscores the value of family interventions in sexual risk prevention. Several studies have demonstrated a link between young people’s sexual behavior and levels of parental monitoring, parent–child communication, and parental discipline. The importance of parental involvement is often underscored in risk-reduction efforts targeted towards adolescents because parents/guardians are in regular contact with their children and, therefore, presumably in a good position to shape and influence young people’s behaviors [8].

According to Sensalire et al. adolescent girls are in a stage of social, psychological, economic, and biological changes [1]. The hardships surrounding the intervention communities such as poverty, GBV further activate adolescent risk lifestyles. The findings show that as interventions addressing specific risk factors unfolded, risk behavior declined. This contextualized and focused interventions have more effect on adolescent sexual behavior in as far as they focus on particular risks appropriate to the target population at a time. This means that interventions for prevention of HIV among adolescents need to focus on specific behaviors prevailing in that community rather than on generalized communication.

Results further indicated a decline in unwanted teenage pregnancy at follow up since all AGWY who were pregnant desired to have the pregnancy at the time they conceived compared to the baseline. The results give rise to two important considerations. First, AGYW who were pregnant are at high risk of HIV by virtue of unprotected sex. Secondly the findings verify low contraceptive use.

As the paper notes, everyone was a year older and a third of them were married at the end of the study. Perhaps the change in sexual behavior of AGYW following the roll out of the QBC model was reinforced with marriage or the more rational decision making that comes with maturity. However, this would only apply to a small proportion of those who were married implying that the majority of AGYW sexual behavior was due to interventions related with QBC model, other factors constant.

The QBC model utilized a participatory approach and involved various community resource persons including peers to address risks associated with HIV. These were known as QI teams. The decline in risky sexual behavior, namely, reduction in multiple sexual partnerships, and transactional sex among AGYW, within a relatively short period of time implies that behavioral interventions involving the community are effective, possibly because of the close proximity to the target group and the general understanding of the socio-cultural factors that drive infection. Broad-based community participation in a behavioral intervention facilitates the selection and use of culturally appropriate strategies to reach the target audience.

The study had some limitations. First, it is unclear whether similar processes exist for boys since the sample was comprised entirely of girls. Second, our study applied to a longitudinal sample but without a control group. Thus, it’s in some way unknown whether other factors could have contributed to changes in sexual behavior. Although this study provides some evidence of change of sexual behavior following the roll out of the quality improvement for behavior change model (QBC), the study did not test for sustainable change of the desired behavior in the long run given the short study duration. All the behaviors are self-reported. It could be that the participants learned quite clearly during the intervention what they should be doing and may have given the response they thought the interviewers wanted to hear. However this was minimized through an independent assessment of the cohort’s sexual behavior and the use of multiple checks in the data collection tools.

In light of these limitations, future research should aim to examine QBC models for girls and boys and with a control group to rule out existence of other factors that could influence short term changes in sexual behavior. The longitudinal study designs should provide reasonable time to determine sustainable behavior change.

## Conclusion

Scale-up of the QBC intervention to reduce or prevent HIV infection among AGYW should be supported because it offers a feasible solution within the reach of the target population. Its success however depends on use of a participatory approach and involvement of the community. Therefore, interventions aimed at reducing or preventing HIV infection among AGYW should involve peers and respected members of the community working as a team. Quality improvement approach is a fundamental tool in as far as the change strategies and activities are developed and implemented in a way that is appropriate to the target groups and behaviors of interest.

## Abbreviations

AGYW: adolescent girls and young women
ANOVA: analysis of variance
ASSIST: applying science to strengthen and improve systems
DREAMS: determined resilient empowered AIDS-free mentored and safe
GBV: gender based violence
HCT: HIV counseling and testing
HIV: Human Immunodeficiency Virus
IRB: Institutional Review Board
LLC: Limited Liability Company
LRA: Lord’s Resistance Army
PEPFAR: President’s Emergency Plan for AIDS Relief
QBC: quality improvement for behavioral change
QI: quality improvement
REC: Research Ethics Committee
SDS: strengthening decentralization for sustainability
STIs: sexually transmitted infections
UNCST: Uganda National Council of Science and Technology
USAID: United States Agency for International Development
VHTs: Village Health Teams
VMMC: voluntary medical male circumcision
